# Association of smoking and physical inactivity with MRI derived changes in cardiac function and structure in cardiovascular healthy subjects

**DOI:** 10.1038/s41598-019-54956-8

**Published:** 2019-12-09

**Authors:** Anina Schafnitzel, Roberto Lorbeer, Christian Bayerl, Hannah Patscheider, Sigrid D. Auweter, Christa Meisinger, Margit Heier, Birgit Ertl-Wagner, Maximilian Reiser, Annette Peters, Fabian Bamberg, Holger Hetterich

**Affiliations:** 1 Department of Radiology, Ludwig-Maximilians-University Hospital, Marchioninistr. 15, 81377 Munich, Germany; 2 Institute of Epidemiology II, Helmholtz Zentrum München, Ingolstädter Landstraße 1, 85764 Neuherberg, Germany; 3 Center for Diagnostic and Therapeutic Radiology, Department of Diagnostic and Interventional Radiology, Medical Center - University of Freiburg, Faculty of Medicine, Hugstetter Str. 55, 79106 Freiburg, Germany; 4 Chair of Epidemiology, Ludwig-Maximilians-University Munich, Geschwister-Scholl-Platz 1, 80539 Munich, Germany; 5 UNIKA-T Augsburg, Neusaesser Str. 47, 86156 Augsburg, Germany

**Keywords:** Epidemiology, Risk factors

## Abstract

We aimed to investigate the association of smoking and physical exercise on ventricular function and structure, determined by cardiac magnetic resonance imaging (CMR), in subjects without known cardiovascular diseases. A total of 381 participants (median age 57 years) of the Cooperative Health Research in the Region of Augsburg (KORA) FF4 cohort underwent CMR. The participants’ smoking and sporting habits were measured by a questionnaire. Physical inactivity was associated with a reduction of left ventricular ejection fraction (LV-EF), stroke volume, early diastolic peak filling rate and peak ejection rate of the left ventricle as well as right ventricular stroke volume. LV-EF was reduced in subjects with almost no physical activity compared to subjects with regular physical activity (68.4%, 95%CI 66.8–70.1% vs. 70.8%, 95%CI 69.2–72.3%, p < 0,05). Smokers had lower right ventricular end-diastolic volumes (80.6 ml/m², 95%CI 76.7–84.5 ml/m²; never-smokers: 85.5 ml/m², 95%CI 82.6–88.3 ml/m²; p < 0.05) but higher extracellular volume fractions (ECV) and fibrosis volumes (34.3 ml, 95%CI 32.5–36.0 ml, vs. 31.0 ml, 95%CI 29.6–32.3 ml, p < 0.01). We conclude that asymptomatic individuals without known cardiovascular diseases show differences in cardiac function and structure depending on their physical activity and smoking habits. This underlines the importance of prevention and health education.

## Introduction

Deaths due to cardiovascular disease increased by 12.5% globally between 2005 and 2015^1^. Smoking and physical inactivity are among the main modifiable risk factors^2–4^. In several clinical and population-based studies, smoking and physical inactivity were independently associated with advanced cardiovascular disease such as heart failure, myocardial infarction and stroke as well as cardiovascular mortality^5–12^. The prevalence of smoking declined in the last 25 years, but still about 21% of men and 6% of women worldwide were smoking in 2015^10^. More than 35% of adults have a sedentary life style with low physical activity^10^.

Some studies have also linked smoking and physical inactivity to reduced myocardial function and reduced ventricular mass^13–16^. However, there is only limited data on the association of smoking and physical inactivity on subclinical cardiovascular changes in asymptomatic individuals without a history of cardiovascular disease. Even less data is available about the influence of cardiac risk factors on diffuse myocardial fibrosis or myocardial scarring in asymptomatic individuals.

Cardiac magnetic resonance imaging (CMR) is the standard of reference for the evaluation of right ventricular function and is less patient- and investigator-depended than echocardiography concerning left ventricular structure and function^17–19^. Furthermore, it allows to evaluate myocardial scarring and diffuse fibrosis^20^.

Thus, we aimed to investigate if smoking and physical inactivity are associated with changes in cardiac structure and function as determined by CMR in asymptomatic individuals without known cardiovascular diseases.

We hypothesized that smoking and physical inactivity are independent risk factors for elevated left and right ventricular mass, decreased left and right ventricular function and increased amounts of myocardial fibrosis and scarring.

## Results

### Study population

Out of the 400 participants in our sub-study, a total of 381 participants were included in the analysis. Nineteen examinations had to be excluded because for poor image quality or missing left ventricular data. The right ventricular data was available in 337 subjects. In 35 cases, data were incomplete due to software problems during the MRI scan or because the examination was aborted by the study participant. For 28 subjects, short axis images did not completely include the basal part of the right ventricle and were therefore excluded from the final evaluation. Data of 260 subjects were included in the calculation of extracellular volume fraction (ECV) and 257 subjects in the fibrosis calculation, the others had to be excluded due to artifacts, incomplete measurements or missing haematocrit levels. In 368 subjects, Late Gadolinium Enhancement (LGE) measurements could be analysed – 32 examinations had to be discarded due to missing data or poor image quality. Baseline characteristics of the study population are shown in Table 1. The study population consisted of 162/381 (43%) women and 219/381 (58%) men. Diabetes and hypertension were found in 49/381 (13%) and 127/381 (33%) of subjects, respectively. In general men had a more pronounced cardiovascular risk profile. The majority of participants reported regular physical activity of at least 1 h/week (227/381, 60%). A minority were active smokers (76/381, 20%), while most participants were former or never smokers (167/381, 44% and 138/381, 36%). Men were more often smokers or ex-smokers (148/381, 68%) and physically inactive (65/381, 30%) compared to women (95/381, 59% and 34/381, 21%, respectively). Medication included antihypertensive drugs (94/381, 24.6%), lipid-lowering (40/381, 10.5%) and antidiabetic medication (29/381, 7.6%).

**Table 1:** Baseline characteristics of the study sample (N = 381).

	Women	Men
	N = 162	N = 219
Age (years)	58 (48; 64)	56 (49; 64)
**Smoking status**		
Never-smoker	67 (41.4%)	71 (32.4%)
Ex-smoker	60 (37.0%)	107 (48.9%)
Current smoker	35 (21.6%)	41 (18.7%)
Smoking Pack Years	1.1 (0; 11.7)	4.5 (0; 26.0)
**Physical activity**		
Regular >2 h/week	45 (27.8%)	64 (29.2%)
Regular 1 h/week	58 (35.8%)	60 (27.4%)
Irregular 1 h/week	25 (15.4%)	30 (13.7%)
(Almost) no activity	34 (21.0%)	65 (29.7%)
Body mass index (kg/m²)	26.8 (23.1; 30.9)	27.9 (25.3; 30.7)
Body surface area (m^2^)	1.8 (1.7; 1.9)	2.1 (2.0; 2.2)
Systolic blood pressure (mmHg)	113 (103; 122)	126 (115; 136)
Diastolic blood pressure (mmHg)	72 (66; 77)	78 (71; 83)
Hypertension	46 (28.4%)	81 (37.0%)
**Diabetes status**		
Non-Diabetics	113 (69.8%)	119 (54.3%)
Pre-Diabetics	36 (22.2%)	64 (29.2%)
Diabetics	13 (8.0%)	36 (16.4%)
HDL-C (mg/dl)	68 (57; 82)	54 (45; 64)
LDL-C (mg/dl)	134 (112; 157)	140 (117; 162)
TG (mg/dl)	95 (68; 121)	122 (85; 186)
Antihypertensive medication	44 (27.2%)	50 (22.8%)
Lipid-lowering medication	18 (11.1%)	22 (10.0%)
Antidiabetic medication	11 (6.8%)	8.2%)

Data are given as number (percentage) or median (25^th^ and 75^th^ percentile).

HDL-C, high-density-lipoprotein cholesterol; LDL-C, low-density-lipoprotein cholesterol; TG, triglycerides.

### Cardiac function and structure

Overall, mean left and right ventricular parameters of the study population were within normal limits (left ventricular end-systolic volume (LV-ESV): 40.7 ± 18.1 ml, left ventricular end-diastolic volume (LV-EDV): 129.1 ± 32.9 ml, left ventricular ejection fraction (LV-EF): 69.2 ± 8.2%, left ventricular stroke volume (LV-SV): 88.3 ± 20.7 ml, left ventricular (LV) myocardial mass: 140.7 ± 35.0 g, right ventricular end-diastolic volume (RV-EDV): 165.5 ± 39.8 ml, right ventricular end-systolic volume (RV-ESV): 79.1 ± 25.9 ml, right ventricular stroke volume (RV-SV): 86.4 ± 19.5 ml and right ventricular ejection fraction (RV-EF): 52.8 ± 7.0%) with some subjects with reduced LV-EF (8/381, 2%) or RV-EF (31/381, 8%) or increased LV volumes (5/381, 1%) or RV volumes (15/381, 4%)^21^. A total number of 11 individuals (2.9%) showed LGE and mean ECV and fibrosis volume were 0.24 ± 0.03% and 31.8 ± 8.2%, respectively.

### Association of physical inactivity with cardiac function and structure

**Figure 1:**
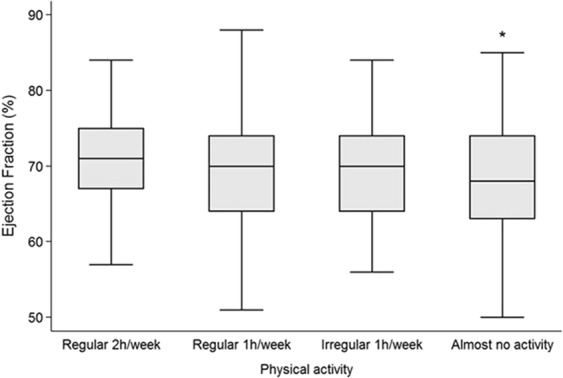
Observed ejection fraction in different groups of physical activity demonstrated by box plots (box = interquartile range, horizontal line = median, whiskers = lower and upper adjacent values), (*p < 0.05, reference category = “regular 2 h/week”). Subjects with regular physical activity 2 h/week showed significantly higher LV-EF than subjects with (almost) no physical activity.

Multivariable linear regression analysis revealed that physical inactivity was associated with a decrease in LV-SV, LV-EF, Peak Ejection Rate (PER) and early diastolic filling rate (PFR1). There was a decrease in LV-EF from subjects with regular 2 h exercise per week (70.8%, 95%-CI 69.2–72.3%) to subjects with almost no exercise (68.4%, 95%-CI 66.8–70.1%) (p = 0.044). All LV-EF values of the different physical activity groups are displayed in Fig. 1. LV-SV was lower in the inactive group (44.1 ml, 95%-CI 42.3–46 ml) compared to the most active group (47.5 ml, 95%-CI 45.8–49.3 ml) (p = 0.009). The inactive group had the lowest PER (335.8 ml/s, 95%-CI −361.2–310.4 ml/s) while the most active group had the highest PER (374.6 ml/s, 95%-CI −398.7–−350.5 ml/s) (p = 0.033). The myocardial relaxation measured by PFR1 was significantly higher in the most active group (254.2 ml/s, 95%-CI 234.4–274.0 ml/s) compared to the inactive group (204.8 ml/s, 95%-CI 183.9–225.7 ml/s) (p = 0.001). There were no significant associations between physical inactivity and LV-EDV, LV-ESV, late diastolic filling rate (PFR2) or myocardial mass (Table 2). RV-SV was significantly lower in the inactive group (43.6 ml, 95%CI 41.8–45.4 ml) compared to the most active group (46.6 ml, 95%CI 44.9–48.4 ml) (p = 0.020). There were no significant associations between physical inactivity and RV-EDV, RV-ESV or RV-EF (Table 3) or between physical activity and the amount of LGE or fibrosis volume and ECV (Table 4).

**Table 2:** Physical activity and left ventricular volumes, myocardial mass and function (N = 381).

	Physical activity			
	Regular >2 h/week^§^	Regular 1 h/week	Irregular 1 h/week	(Almost) no activity
	N = 109	N = 118	N = 55	N = 99
End-diastolic volume/BSA (ml/m^2^)	67.9 (65.2; 70.6)	65.6 (63.1; 68.2)	66.4 (62.6; 70.2)	65.0 (62.2; 67.9)
End-systolic volume/BSA (ml/m^2^)	20.3 (18.7; 21.9)	21.0 (19.5; 22.5)	21.1 (18.9; 23.4)	21.0 (19.3; 22.7)
Stroke volume/BSA (ml/m^2^)	47.5 (45.8; 49.3)	44.6 (43.0; 46.3)*	45.3 (42.9; 47.7)	44.1 (42.3; 46.0)**
Ejection Fraction (%)	70.8 (69.2; 72.3)	68.7 (67.3; 70.2)	68.5 (66.4; 70.7)	68.4 (66.8; 70.1)*
Myocardial mass, diastolic (g)	143.6 (139.0; 148.3)	138.6 (134.2; 143.0)	140.8 (134.3; 147.3)	140.0 (135.1; 144.9)
Myocardial mass, systolic (g)	146.6 (141.7; 151.4)	141.6 (137; 146.2)	141.3 (134.5; 148.1)	142.7 (137.6; 147.8)
Peak systolic ejection rate (ml/s)	−374.6 (−398.7; −350.5)	−346.0 (−368.8; −323.1)	−363.8 (−397.8; −329.8)	−335.8 (−361.2; −310.4)*
Early diastolic filling rate (ml/s)	254.2 (234.4; 274.0)	218.0 (199.2; 236.7)**	223.5 (195.6; 251.5)	204.8 (183.9; 225.7)**
Late diastolic filling rate (ml/s)	253.2 (226.7; 279.7)	237.8 (212.7; 262.9)	237.4 (199.9; 274.8)	217.9 (189.9; 245.8)

Data are adjusted predicted means and 95% confidence intervals after linear regression adjusted for age, sex, body mass index, systolic blood pressure, diastolic blood pressure, diabetes mellitus; BSA, body surface area.

**p < 0.01, *p < 0.05, ^§^Reference category = “regular >2 h/week”.

**Table 3:** Physical activity and right ventricular volumes and function (N = 337).

	Physical activity			
	Regular >2 h/week^§^	Regular 1 h/week	Irregular 1 h/week	(Almost) no activity
	N = 97	N = 107	N = 47	N = 86
End-diastolic volume/BSA (ml/m^2^)	87.4 (84.1; 90.6)	83.1 (80.0; 86.2)	86.1 (81.4; 90.8)	83.2 (79.7; 86.7)
End-systolic volume/BSA (ml/m^2^)	40.8 (38.5; 43.0)	40.3 (38.2; 42.4)	41.2 (38.0; 44.4)	39.6 (37.3; 42.0)
Stroke volume/BSA (ml/m^2^)	46.6 (44.9; 48.4)	42.9 (41.3; 44.5)**	44.9 (42.4; 47.4)	43.6 (41.8; 45.4)*
Ejection Fraction (%)	53.8 (52.5; 55.1)	52.0 (50.8; 53.3)	52.9 (51.0; 54.8)	52.7 (51.3; 54.1)

Data are adjusted predicted means and 95% confidence intervals after linear regression adjusted for age, sex, body mass index, systolic blood pressure, diastolic blood pressure, diabetes mellitus; BSA = body surface area.

**p < 0.01, *p < 0.05, ^§^Reference category = “regular 2 h/week”.

**Table 4:** Physical activity and left ventricular scarring and fibrosis.

	Physical activity			
	Regular >2 h/week^§^	Regular 1 h/week	Irregular 1 h/week	(Almost) no activity
ECV (%)	0.24 (0.23; 0.25)	0.24 (0.24; 0.25)	0.25 (0.24; 0.26)	0.24 (0.24; 0.25)
Fibrosis volume (ml)	32.6 (31.0; 34.1)	31.2 (29.8; 32.6)	32.3 (30.2; 34.5)	31.4 (29.6; 33.1)
LGE^#^ (%)	3.8 (0.1; 7.5)	2.0 (−0.8; 4.8)	2.1 (−2.0; 6.2)	3.2 (0.1; 6.2)

Data are adjusted predicted means or ^#^proportions and 95% confidence intervals after linear regression or ^#^logistic regression adjusted for age, sex, body mass index, systolic blood pressure, diastolic blood pressure, diabetes mellitus; ECV = extracellular volume fraction; LGE = Late Gadolinium Enhancement.

All p > 0.05. ^§^Reference category = “regular >2 h/week”.

### Association of smoking status with cardiac function and structure

There were no significant associations between smoking status and left ventricular function (Table 5). However, there was a tendency of increased LV mass in current smokers (145.2 ml 95%-CI 139.8–150.7 ml) compared to never-smokers (139.6 ml, 95%-CI 135.5–143.6 ml) (p = 0.102).

**Table 5:** Smoking status and left ventricular myocardial mass and function (N = 381).

	Smoking status		
	Never-smoker§	Ex-smoker	Current smoker
	N = 138	N = 167	N = 76
End-diastolic volume/BSA (ml/m^2^)	66.1 (63.7; 68.4)	67.1 (64.9; 69.2)	64.7 (61.6; 67.9)
End-systolic volume/BSA (ml/m^2^)	21.1 (19.7; 22.5)	20.9 (19.6; 22.2)	20.2 (18.3; 22.0)
Stroke volume/BSA (ml/m^2^)	45.0 (43.4; 46.5)	46.2 (44.8; 47.6)	44.6 (42.5; 46.6)
Ejection Fraction (%)	68.8 (67.4; 70.1)	69.5 (68.3; 70.8)	69.3 (67.5; 71.1)
Myocardial mass, diastolic (g)	139.6 (135.5; 143.6)	139.6 (135.9; 143.3)	145.2 (139.8; 150.7)
Myocardial mass, systolic (g)	142.2 (138.0; 146.5)	141.7 (137.8; 145.5)	148.7 (143.0; 154.4)
Peak systolic ejection rate (ml/s)	−353.4 (−374.6; −332.3)	−358.7 (−377.9; −339.4)	−345.0 (−373.5; −316.5)
Early diastolic filling rate (ml/s)	226.8 (209.3; 244.4)	228.9 (213.0; 244.9)	216.7 (193.1; 240.3)
Late diastolic filling rate (ml/s)	240.2 (217.0; 263.4)	241.3 (220.2; 262.4)	221.7 (190.5; 252.9)

Data are adjusted predicted means and 95% confidence intervals after linear regression adjusted for age, sex, body mass index, systolic blood pressure, diastolic blood pressure, diabetes mellitus; BSA = body surface area.

All p > 0.05. ^§^Reference category = “never-smoker”.

RV-EDV was significantly lower in current smokers (80.6 ml, 95% CI 76.7–84.5 ml) than in never-smokers (85.5 ml, 95% CI 82.6–88.3 ml) (p = 0.048). Smoking status showed no significant associations with RV-ESV, RV-SV and RV-EF (Table 6).

**Table 6:** Smoking status and right ventricular function (N = 337).

	Smoking status		
	Never-smoker§	Ex-smoker	Current smoker
	N = 121	N = 149	N = 67
End-diastolic volume/BSA (ml/m^2^)	85.5 (82.6; 88.3)	86.1 (83.5; 88.7)	80.6 (76.7; 84.5)*
End-systolic volume/BSA (ml/m^2^)	41.3 (39.4; 43.2)	40.6 (38.9; 42.4)	38.1 (35.5; 40.7)
Stroke volume/BSA (ml/m^2^)	44.2 (42.7; 45.7)	45.5 (44.1; 46.9)	42.5 (40.5; 44.6)
Ejection Fraction (%)	52.1 (50.9; 53.3)	53.2 (52.2; 54.3)	53.4 (51.8; 54.9)

Data are adjusted predicted means and 95% confidence intervals after linear regression adjusted for age, sex, body mass index, systolic blood pressure, diastolic blood pressure, diabetes mellitus; BSA = body surface area.

*p < 0.05, ^§^reference category = “never-smoker”.

Smokers showed significantly higher ECV (0.25%, 95%CI 0.24–0.26% vs. 0.24%, 95%CI 0.24–0.26%, p < 0.01) and fibrosis volumes (34.3 ml, 95%CI 32.5–36.0 ml, vs. 31.0 ml, 95%CI 29.6–32.3 ml, p < 0.01) (Table 7).

**Table 7:** Smoking status and left ventricular scarring and fibrosis.

	Smoking status		
	Never-smoker§	Ex-smoker	Current smoker
ECV (%)	0.24 (0.23; 0.24)	0.24 (0.23; 0.24)	0.25 (0.24; 0.26)**
Fibrosis volume (ml)	31.0 (29.6; 32.3)	31.3 (30.0; 32.5)	34.3 (32.5; 36.0)**
LGE (%)^#^	3.0 (0.2; 5.8)	3.9 (1.3; 6.4)	0.6 (−3.2; 4.5)

Data are adjusted predicted means or ^#^proportions and 95% confidence intervals after linear regression or ^#^logistic regression adjusted for age, sex, body mass index, systolic blood pressure, diastolic blood pressure, diabetes mellitus; ECV = extracellular volume fraction; LGE = Late Gadolinium Enhancement.

**p < 0.01. ^§^Reference category = “never-smoker”.

## Discussion

In this cross-sectional study, we examined the association of physical inactivity and smoking habits on cardiac function and structure as assessed by CMR in subjects without known cardiovascular disease. Parameters for left and right ventricular function were within normal limits for the vast majority of participants. However, we found that physical inactivity is associated with a reduction of LV and RV systolic and LV diastolic function. Furthermore, smoking was associated with a reduction of RV-EDV and with elevated ECV and fibrosis volumes.

These findings are in line with results of the Multi-Ethnic Study of Atherosclerosis (MESA)^16^. In MESA as well as in our study, LV-SV increased with exercise. In contrast to MESA, we have also found a positive effect on LV-EF. Potentially these results could be explained by differences in the assessment of physical activity or differences in the ethnicities of the study (the KORA population being completely Caucasian). Furthermore, we used a 3-Tesla MRI system, which offers an increased signal-to-noise-ratio and contrast-to-noise-ratio compared to 1.5-Tesla^22^.

Levy *et al*. showed that older and untrained subjects had a lower PFR1^23^. This correlates with our results, where subjects with the highest physical activity per week had the highest PFR1. In our study subjects with more than 2 hours of physical activity per week had the highest PER, as was already demonstrated by Stratton *et al*.^24^, where untrained men had lower PER.

Our study shows that physical activity has a significant positive effect on RV-SV. Regular physical activity also positively influenced RV-EDV and RV-EF, but these results were not significant. This is in line with the results of Aaron *et al*. in the MESA trial^15^. The higher RV-SV could be caused by hypertrophy of cardiomyocytes due to physical activity, causing higher left and right SV^25^.

In former studies, subjects with higher physical activity rates showed no difference in cardiac fibrosis volumes^26^, but more LGE^27^, while in our study physical activity had no significant effect on fibrosis volumes, ECV or LGE. As physical activity influences cardiac function and hypertrophy, fibrosis and ECV could also be affected, whereas presence of LGE should not differ between subjects with more or less physical activity. These partly differing results need more research in the future.

We observed a trend towards increased left ventricular mass in smokers, a result that is in line with MESA and other studies^16,28^. In contrast, the LARGE study found a smaller left ventricular mass in smokers, maybe due to the special cohort of the LARGE study, which comprised only healthy young men^29^.

Analogous to the MESA study, RV-EDV was lower in smokers^30–32^. Cigarette smoke includes several toxic substances that cause aging of cardiomyocytes and thus reduced compliance of the right ventricle^33^. Smoking can lead to chronic obstructive pulmonary disease, which causes hyperinflation of the lungs^32^. Both mechanisms can thus reduce RV filling.

We were able to demonstrate that current smokers had significantly higher ECV and fibrosis volumes compared to non-smokers, which can be explained by oxidative stress and inflammation causing cardiac remodeling in smokers^34^. In MESA, smokers also had more LGE^35^, whereas in our study smokers had less LGE, but this result was not significant. It is known that smokers are more vulnerable concerning coronary heart disease so it could be expected that smokers and ex-smokers show more LGE than never-smokers. This, too, needs further research with larger cohorts.

### Limitations and strengths of the study

As this is a cross-sectional study, we can only show correlations; it is not possible to draw conclusions about causations. As opposed to the LARGE study (young white males) or the MESA study (4 racial/ethnic groups) our subjects were aged 38–72, caucassian, and were approximately 50% females. As a result, our cohort is better suited for the population in Germany than the LARGE or MESA cohorts. Our results are in general in line with the results of other large cohort studies examining subclinical cardiovascular changes depending on risk factors.

Smoking status was assessed via questionnaire, so there was no biochemical confirmation of smoking status. Whereas subjects with known cardiovascular diseases were excluded from the KORA study, participants with known pulmonary diseases that could affect the right ventricle, were not excluded. Another limitation is that the subjects’ amount of physical activity was based on an interview and not validated via ergometry, resting heart rate or electronic activity trackers like wristbands.

The differences between the examined groups were small and most probably have no clinical significance but may represent early manifestations of potentially progressive disease.

Most studies about the impact of cardiac risk factors used echocardiography, for example the Framingham Study. Echocardiography is a widely available method, it is fast, cost-effective and does not use radiation. Unfortunately, echocardiography is more dependent on the patient’s constitution, depending on the patient’s acoustic windows, and has quite a high inter-observer variability^36^. Especially when measuring LV-EF using the Simpson method, echocardiography has its limitations because frequently the endocardial contours are often not clearly visible when not using contrast agents^17^. Whereas cardiac magnetic resonance imaging (CMR) has lower inter-observer variabilities, is less dependent on acoustic windows and shows better contrast between endocardium and blood in order to properly measure endocardial contours to measure left ventricular volumes and calculate left ventricular ejection fraction^18,37^. For left ventricular hypertrophy, CMR has higher sensitivity rates than echocardiography^38^. Furthermore, it was shown that CMR has higher reproducibility rates in right ventricular parameters^39,40^. CMR can therefore be called the standard of reference for detecting the smallest, but still significant changes in cardiac function and mass parameters^19^.

## Conclusions

Using CMR, our study showed that in subjects without a history of cardiovascular diseases, parameters of left and right ventricular function increase with physical activity and decrease with smoking. In addition, we were found increases in ECV and fibrosis volumes in smokers. These results underline once more the importance of prevention and health education in order to prevent subclinical and in hereafter symptomatic cardiovascular changes.

## Methods

### Study population

Study participants were recruited from the second follow-up (FF4) of the population-based KORA (“*Kooperative Gesundheitsforschung in der Region Augsburg*”, Cooperative Health research in the region of Augsburg) S4 cohort. S4 is a health survey (1999–2001) comprising 4,261 individuals, 25 to 74 years of age, in the region of Augsburg in the south of Germany. The first follow-up (F4) was conducted between 2006 and 2008 with 3,080 participants and the second follow-up (FF4) took place in 2013/2014 with 2,279 participants^41^.

The current study population was drawn from a sub-study that aimed at examining MRI-derived subclinical cardiovascular disease burden in diabetics, pre-diabetics and controls without a history of cardiovascular disease^42^. For this study, subjects were recruited from the KORA FF4 follow-up. Exclusion criteria were: age >72 years, subjects with validated/self-reported stroke, myocardial infarction or revascularization, subjects with a cardiac pacemaker or implantable defibrillator, cerebral aneurysm clip, neural stimulator, any type of ear implant, ocular foreign body (e.g. metal shavings), any implanted device (e.g. insulin pump, drug infusion device), pregnant or breast feeding female subjects, claustrophobia, known allergy against gadolinium compounds and serum creatinine ≥1.3 mg/dl. The inclusion criteria were: willingness to undergo whole-body MRI and qualification in the diabetics, prediabetics or control group according to the World Health Organization criteria^43^. In total, 400 subjects aged 38–72 years were included in this study. The study was approved by the institutional review board of the Ludwig-Maximilians-University, Munich, and all participants provided written informed consent^42^. All methods were performed in accordance with the relevant guidelines and regulations.

### MRI examination and evaluation

All images were obtained with the same 3-Tesla MR system (Magnetom Skyra, Siemens Healthcare, Erlangen Germany) in the Institute of Clinical Radiology of the Ludwig-Maximilian University Hospital in Munich. Imaging was performed using an 18-channel body coil in combination with the table-mounted spine matrix coil. The MRI examination was performed within three months after the FF4 visit at the study center. The protocol comprised imaging of the neurocranium, the carotid vessels, liver and whole-body fat imaging as well as cardiac imaging. Total acquisition time was approximately one hour. The full protocol is shown in Supplementary Table S1.

### Cardiac function analysis

Left ventricular function was assessed using cine-steady state free precession sequences which were acquired in four chamber view and short axis stack with 10 slices and 25 phases using the following parameters: slice thickness 8 mm, in-plane voxel size 1.5 × 1.5 mm², field of view 297 × 360 mm², matrix 240 × 160, repetition time 29.97 ms, echo time 1.46 ms, flip angle 63°.

**Figure 2:**
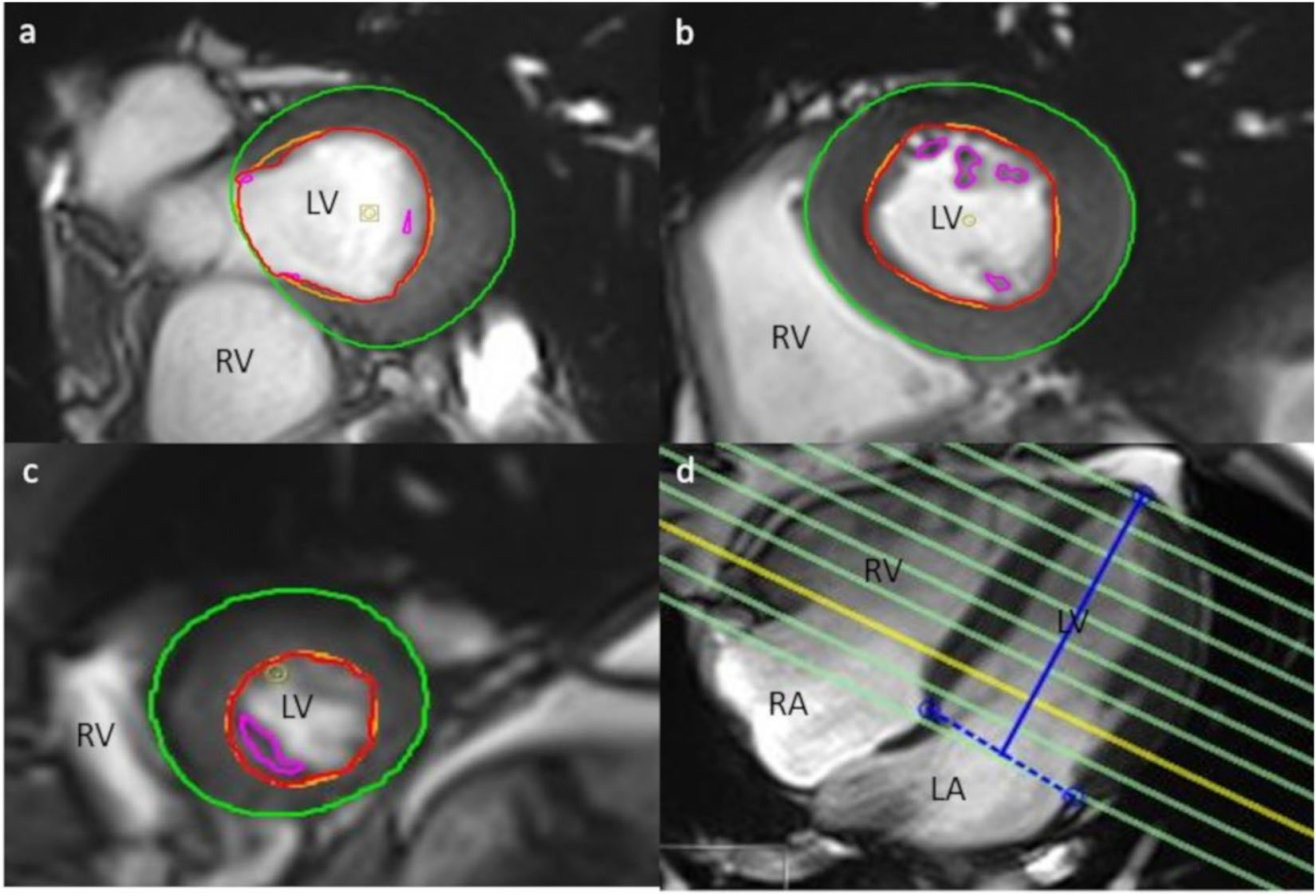
Evaluation of left ventricular function and structure. Basal (**a**), mid-ventricular (**b**), apical (**c**) short axis images and 4-chamber-view (**d**). LV = left ventricle; RV = right ventricle; LA = left atrium; RA = right atrium. Green lines indicate the epicardial contour, red/orange lines the endocardial contour. The blue lines indicate the tagging of the left ventricle on the 4-chamber-view.

Evaluation of cardiac function parameters was performed using a dedicated software package (cvi42, Version 4.1.5(190), Circle Cardiovascular Imaging Inc., Calgary, Canada). The left ventricular short-axis and four chamber view images were analyzed semi-automatically. The reading was performed according to standardized post-processing guidelines of the Society for Cardiovascular Magnetic Resonance^44^ by two readers blinded to any information regarding the subjects’ risk factors and clinical status. After automatic contouring of the endo- and epicardial contours, the outlines were corrected manually, if necessary. The papillary muscles were excluded from the myocardial mass and included in the ventricular volumes. The position of end-diastolic and end-systolic phase were chosen automatically by the software. The derived parameters were: end-systolic volume (LV-ESV), end-diastolic volume (LV-EDV), stroke volume (LV-SV), ejection fraction (LV-EF), systolic and diastolic myocardial mass, cardiac output, and left ventricular wall thickness. An example of the LV contouring is given in Fig. 2. Furthermore, left ventricular filling and ejection rates were determined based on the results of the left ventricular volumetry using an in-house software (pyHeart). Measured parameters were: the peak ejection rate (PER), the peak filling rate of the early diastole (PFR1), the peak filling rate of the late diastole (PFR2) and the delay time between early and late diastolic fillings.

Right ventricular volumes and function were analyzed by manual tracking of the endocardial contour on short-axis and manual detection of the apex and tricuspid valve on four chamber view images according to current guidelines^44^. The largest and smallest right ventricular volume were defined as end-systolic and end-diastolic phase. Papillary muscles were included in the ventricular volume. Parameters included: end-diastolic (RV-EDV) and end-systolic volume (RV-ESV), stroke volume (RV-SV), cardiac output, ejection fraction (RV-EF).

Late gadolinium enhancement (LGE) sequences were acquired 10 minutes after administration of gadopentate dimeglumine (0,2 mmol/kg, Gadovist, Bayer Healthcare, Berlin, Germany) as Fast Low Angle Shot (FLASH) inversion recovery sequences with slice thickness 8 mm, FOV 300 × 360 mm, Matrix 256 × 140, TR 700–1000 ms, TE 1.55 ms and FA 20–55°. Analysis of late gadolinium enhancement (LGE) was performed by two experienced readers using the same software also used for the cardiac function analysis (cvi42; Circle Cardiovascular Imaging). In case of discrepancy, a consensus reading was performed. The presence and distribution pattern (subendocardial, midmyocardial, and epicardial) of LGE was documented using the AHA 17-segment model^45^.

For measuring myocardial fibrosis, T1 ECG-gated steady-state free-precession-based modified Look-Locker inversion-recovery (MOLLI) sequences with 5(3)3 pattern (acquiring 5 images after the first inversion, followed by a 3 heartbeat pause and then again 3 images after the second inversion) were acquired pre- and 10 minutes post contrast. These sequences were acquired on short axis at the mid-ventricular and basal short-axis plane (segments 1–12) with the following parameters:: Slice thickness 8 mm, spatial resolution: 1.5 × 1.5 mm2, acquired voxel size: 2.25 × 1.5 mm2, FOV: 323 × 380 mm using a 256 × 144 mm matrix, TE: 1.1, TI: 100–3500 with a 35° flip angle. T1 relaxation times were calculated per segment (1–12 of the 17 segments of AHA classification). The inner and outer contour of the left ventricular myocardium was segmented following recommendations^46^ to omit influence of surrounding fat or blood by two blinded readers. Segments with obvious artifacts or presence of LGE were discarded. Another region of interest was placed in the blood volume. ECV as a measure of the amount of extracellular matrix was calculated from the T1 relaxation times pre and post contrast by taking into account the haematocrit. Fibrosis volume was derived using the following formula:

- ΔR1myocardium = 1/T1myo-post − 1/T1myo-pre
- ΔR1 blood = 1/T1blood-post − 1/T1blood-pre
- Myocardial partition coefficient (λ)  = (ΔR1myocardium/ΔR1 blood)
- ECV =  (1 − haematocrit level) × λ
- Fibrosis volume = ECV*LVmass/myocardial density [myocardial density = 1.05 g/mL]

### Assessment of smoking habits, physical activity and covariates

Smoking habits and physical activity of the subjects were measured in a standardized interview during the FF4 follow-up at the KORA study center. Physical activity was scaled as follows: Physical activity regularly more than 2 hours per week (group 1), regularly one hour per week (group 2), irregularly one hour per week (group 3), almost no activity (group 4). For smoking the scaling was: smoker, ex-smoker, never-smoker. Subjects were classified as smokers if they reported current regular or sporadic cigarette smoking.

Several co-variates were measured in the KORA FF4 study center by standardized interview, basic health examinations and laboratory analyses. They comprised: age (years), sex (male, female), body mass index (BMI, calculated as weight divided by squared height, kg/m²), waist circumference, body surface area, systolic and diastolic blood pressure, diabetes status (non-diabetics, pre-diabetics, diabetics), intake of antihypertensive, lipid-lowering or antidiabetic medication. Laboratory measurements including HbA1c, glucose, total cholesterol, high and low density lipoprotein and triglycerides were performed according to KORA standards^47^. Blood pressure was measured three times at the right arm of seated participants after an at least five-minute resting period. The mean of the second and third measurement was used for the analyses. Arterial hypertension was defined as increased systolic blood pressure (≥140 mmHg) and/or increased diastolic blood pressure (≥90 mmHg) or use of antihypertensive medication under awareness of having hypertension according to 2003 World Health Organization / International Society of Hypertension criteria^48^.

### Statistical analyses

Baseline characteristics of the study sample were separately described for women and men by median and interquartile range for continuous variables and absolute numbers and percent values for categorical variables.

Associations of physical inactivity and smoking (risk factors = independent variables) with left and right ventricular structure and function parameters (outcomes = dependent variables) were investigated by calculating adjusted predicted outcome means or proportions with 95% confidence intervals by using average co-variable values after separated linear or logistic regression models for each risk factor and each outcome. Adjustments were made for age, sex, body mass index, systolic and diastolic blood pressure as well as diabetes mellitus. Physical inactivity and smoking status were treated as factor variables and each predicted mean was compared with its reference category of regular 2 hours activity per week or never smoking status, respectively. The p-value from t-test of the corresponding β-coefficient from the linear regression model was used to evaluate outcome differences between risk factor groups. Normal distributions of predicted residuals were tested visually. In addition, observed differences of LV-EF between physical activity categories were presented by box plots providing group medians, interquartile ranges and lower and upper adjacent values. All analyses were additionally adjusted for sampling weights considering differences in age, sex and diabetes status between the study sample (n = 400) and the entire KORA cohort (n = 2279, median age = 60 years, 48% men, 15% participants with diabetes) yielding no substantially changed findings. A p-value of <0.05 was considered statistically significant. Statistical analyses were performed using Stata 14.1 (Stata Corporation, College Station, TX, U.S.A.).

## Supplementary information

Supplementary Table S1

## Data Availability

All data are available on the KORA.PASST platform, accessible by contacting the Helmholtz Centre, Munich. Additional information is shown in the appendix.
