# Supplementary material for: Association of smoking and physical inactivity with MRI derived changes in cardiac function and structure in cardiovascular healthy subjects
Source: Sci Rep. 2019 Dec 9;9:18616. doi: 10.1038/s41598-019-54956-8 (PMC6901589; doi:10.1038/s41598-019-54956-8)
Supplement: Supplementary file 1 — Supplementary Table S1 [file 41598_2019_54956_MOESM1_ESM.docx]

# **Association of smoking and physical inactivity with MRI derived changes in cardiac function and structure in cardiovascular healthy subjects**

Anina Schafnitzel^1,3*^, Roberto Lorbeer^1^, Christian Bayerl^1^, Hannah Patscheider^1^, Sigrid D. Auweter^1^, Christa Meisinger^2,4^, Margit Heier^2^, Birgit Ertl-Wagner^1^, Maximilian Reiser^1^, Annette Peters^2^, Fabian Bamberg^1,3^, Holger Hetterich^1^

^1^Department of Radiology, Ludwig-Maximilians-University Hospital, Marchioninistr. 15, 81377 Munich, Germany;

^2^Institute of Epidemiology II, Helmholtz Zentrum München, Ingolstädter Landstraße 1

85764 Neuherberg, Germany;

^3^Center for Diagnostic and Therapeutic Radiology, Department of Diagnostic and Therapeutic Radiology, Medical Center - University of Freiburg, Faculty of Medicine, Hugstetter Str. 55, 79106 Freiburg, Germany;

^4^Chair of Epidemiology, Ludwig-Maximilians-University Munich, Geschwister-Scholl-Platz 1, 80539 Munich, Germany; UNIKA-T Augsburg, Neusaesser Str. 47, 86156 Augsburg, Germany

| **MR-Sequence** | **Weighting / Sequence Type** | **ST (mm)** | **Voxel size, In-plane (mm2)** | **FOV (mm)** | **Matrix** | **TR (ms)** | **TE (ms)** | **TI (ms)** | **Flip angle (°)** |
| --- | --- | --- | --- | --- | --- | --- | --- | --- | --- |
| **Brain** |  |  |  |  |  |  |  |  |  |
| TOF | TOF | 1 | 0.6 x 0.6 | 181 x 200 | 320 x 275 | 20 | 3.43 | N/A | 18 |
| SWI | SWI | 2.5 | 0.9 x 0.9 | 208 x 230 | 256 x 223 | 27 | 20 | N/A | 15 |
| FLAIR | T2, SPACE | 0.9 | 0.5 x 0.5 | 245 x 245 | 256 x 256 | 5000 | 389 | 1800 | 120 |
| **Plaque** |  |  |  |  |  |  |  |  |  |
| T1w carotid plaque | T1w fs | 3 | 0.3 x 0.3 | 165 x 220 | 320 x 240 | 800 | 13 | N/A | 180 |
| **Cardiac Function / Myocardium** | |  |  |  |  |  |  |  |  |
| Cine SAX | SSFP | 8 | 1.5 x 1.5 | 297 x 360 | 240 x 160 | 29.97 | 1.46, 10sl | N/A | 62 |
| Cine LAX | SSFP | 8 | 1.5 x 1.5 | 297 x 360 | 240 x 160 | 29.97 | 1.46 | N/A | 63 |
| MOLLI | T1w | 8 | 1.5 x 1.5 | 323 x 380 | 256 x 144 | 250 - 400 | 1.1 | 100 - 3500 | 35 |
| LGE | FLASH | 8 | 1.4 x 1.4 | 300 x 360 | 256 x 140 | 700 - 1000 | 1.55 | 280 - 345 | 20 - 55 |
| **Hepatic Adipose Content** |  |  |  |  |  |  |  |  |  |
| Dual-echo Dixon | VIBE | 3 | 1.2 x 1.2 | 308 x 380 | 320 x 195 | 4.10 | 1.23; 2.46 | N/A | 9 |
| Multi-echo Dixon | VIBE | 4 | 1.8 x 1.8 | 393 x 450 | 256 x 179 | 8.90 | 1.23; 2.46; 3.69; 4.92; 6.15; 7.38 | N/A | 4 |
| Spectroscopy | STEAM | N/A | 30 x 30 x 30* | N/A | N/A | 3000 | 12.00; 24.00; 36.00; 48.00; 72.00 | N/A | N/A |
| **Body Adiopose Content / Anatomy** | |  |  |  |  |  |  |  |  |
| Dual-echo Dixon | VIBE | 1.7 | 1.7 x 1.7 | 488 x 716 | 256 x 256 | 4.06 | 1.26; 2.49 | N/A | 9 |
| HASTE | T2 | 5 | 1.2 x 1.2 | 296 x 380 | 320 x 200 | 1000 | 91 | N/A | 131 |

**Supplementary Table S1: Cardiovascular Whole-Body MRI Protocol:** TOF: Time of flight, SWI: Susceptibility weighted imaging, FLAIR: Fluid attenuated inversion recovery, T2: T2 weighted, SPACE: Sampling perfection with application optimized contrasts using different flip angle evolution, T1w: T1 weighted, T1w fs: T1 weighted fat saturated, SAX: short axis, LAX: long axis, SSFP: Steady state with free precession, MOLLI: modified look-locker inversion recovery, LGE: Late gadolinium enhancement, FLASH: fast low-angle shot, VIBE: volume interpolated breathhold examination, STEAM: Stimulated echo acquisition method, HASTE: Half fourier acquisition single shot turbo spin echo. *voxel size.
